# EXTENDING HOLISTIC, MULTIDISCIPLINARY SLEEP HEALTH INTERVENTION TO OVERLOOKED GROUPS OF OLDER ADULTS

**DOI:** 10.1093/geroni/igad104.0667

**Published:** 2023-12-21

**Authors:** Stacey Schepens Niemiec

**Affiliations:** University of Southern California, Los Angeles, California, United States

## Abstract

Sleep health and overall well-being across the lifespan are inextricably linked. Sleep difficulties are a common occurrence in older adulthood, with disturbed sleep ranking only second to pain as a problematic symptom interfering with older peoples’ daily lives. Exacerbating the issue are sleep health disparities such that sleep disturbances disproportionately affect members of under-represented, marginalized, and resource-poor communities. Building, implementing, and mobilizing knowledge from sleep-promoting interventions with potential to effect change on sleep health and quality of life disparities are urgently needed. From an occupational therapy lens, “rest and sleep” is classified as one of nine fundamental “occupations”—other more widely recognized occupations include those such as ADL and work. The Person, Environment, Occupation-Performance (PEOP) Model, an occupation-centric guiding framework that considers multiple layers of dynamic and interacting factors pertinent to individuals’ occupational performance, has been applied to sleep intervention. From this model, one can appreciate the myriad facilitators of and barriers to realizing healthy and restorative rest and sleep, directing attention to a wide range of relevant issues such as cultural practices, societal attitudes, and resource scarcity. This presentation will overview the need for addressing inequities in sleep health and extending socio-culturally responsive services to traditionally overlooked subgroups of older people. Examples will be provided of holistic, multidisciplinary intervention research and programming that work to transform the landscape of sleep health disparities in later life.

